# Clinical Utility of the Apolipoprotein A2 Isoform Index as a Tumor Marker for Pancreatic Cancer

**DOI:** 10.1002/jgh3.70375

**Published:** 2026-02-26

**Authors:** Takashi Hoshino, Masafumi Mizuide, Yuhei Suzuki, Hidetoshi Yasuoka, Atsushi Naganuma, Takeshi Hatanaka, Satoru Kakizaki, Shomei Ryozawa

**Affiliations:** ^1^ Department of Gastroenterology NHO Takasaki General Medical Center Takasaki Japan; ^2^ Department of Gastroenterology Saitama Medical University International Medical Center Hidaka Japan; ^3^ Department of Gastroenterology Gunma Saiseikai Maebashi Hospital Maebashi Japan; ^4^ Department of Clinical Research NHO Takasaki General Medical Center Takasaki Japan

**Keywords:** apolipoprotein A2 isoform index, CA19‐9, Lewis antigen, pancreatic cancer, tumor marker

## Abstract

**Background and Aims:**

We investigated the clinical utility of the apolipoprotein A2 isoform index (apoA2‐i index), a novel tumor marker for pancreatic cancer, in clinical practice, particularly for CA19‐9‐negative pancreatic cancer.

**Methods:**

Between May 2024 and July 2025, patients who underwent abdominal computed tomography (CT) for the differential diagnosis of pancreatic diseases had their CEA, CA19‐9, and apoA2‐i indexes measured. We evaluated the sensitivity and specificity for pancreatic cancer and analyzed the relationship between the apoA2‐i index and pancreatic atrophy or main pancreatic duct dilatation on CT.

**Results:**

A total of 115 patients were included: 38 with pancreatic cancer, 47 with intraductal papillary mucinous neoplasms (IPMNs), and 30 with other conditions. The median age was 72.0 years, and 55 patients (47.8%) were male. The sensitivity of apoA2‐i index for pancreatic cancer was 65.8%, while the specificity was 85.7%. The combined use of CA19‐9 and the apoA2‐i index increased sensitivity to 78.9% (specificity, 81.8%). Among 17 patients with CA19‐9‐negative pancreatic cancer, 9 (52.9%) tested positive for the apoA2‐i index. The positive rates for stage 0/I pancreatic cancer were 12.5% for CA19‐9 and 62.5% for the apoA2‐i index. Regarding CT findings, the apoA2‐i index significantly decreased with the progression of pancreatic atrophy (*p* = 0.027) and was also significantly lower in patients with pancreatic duct dilatation (*p* = 0.016).

**Conclusions:**

The apoA2‐i index can detect CA19‐9‐negative pancreatic cancer, and the combination of the apoA2‐i index with CA19‐9 enhances diagnostic performance in clinical practice.

## 1 Introduction

Pancreatic cancer is one of the leading causes of cancer‐related deaths worldwide and carries one of the poorest prognoses among gastrointestinal malignancies [1]. In Japan, it is the third most common cause of cancer death, with age‐adjusted incidence and mortality steadily increasing over recent decades [2]. Although early‐stage diagnosis and surgical resection have improved outcomes [1, 3], most cases are already advanced and inoperable at the time of diagnosis. Because early‐stage pancreatic cancer is often asymptomatic, delayed diagnosis remains a major problem, highlighting the urgent need for reliable tools for early detection, including effective tumor markers.

CA19‐9 is the most widely used tumor marker in clinical practice [4], but it has important limitations. False‐positive elevations occur in benign conditions such as biliary obstruction, cholangitis, and liver disease [5]. In addition, some patients have CA19‐9‐negative pancreatic cancer, partly due to Lewis antigen deficiency because CA19‐9 synthesis depends on Lewis antigen expression; genetically Lewis antigen–negative individuals (approximately 5%–10% of the Japanese population) may not show elevated levels even in the presence of cancer [6]. Furthermore, CA19‐9 production may vary according to tumor differentiation and cellular characteristics, even in Lewis antigen‐positive individuals.

These shortcomings underscore the need for novel tumor markers to complement CA19‐9 and for diagnostic algorithms incorporating multiple markers. Honda et al., reported the apolipoprotein A2 isoform index (apoA2‐i index) as a promising biomarker for pancreatic cancer [7, 8, 9, 10]. Abnormal C‐terminal processing of circulating apoA2 homodimers, particularly apoA2‐I, has been observed in patients with pancreatic cancer and in high‐risk individuals [7, 8, 9, 10]. Pancreatic exocrine dysfunction causes aberrant apoA2 processing, resulting in reduced APOA2‐AT/TQ (apoA2‐i index) levels in affected patients [7, 8, 9, 10]. The apoA2‐i index was approved as a tumor marker in Japan in March 2024 and has since been introduced into clinical practice; however, real‐world evidence remains limited.

This study aimed to assess the clinical utility of the apoA2‐i index in routine practice, particularly in patients with CA19‐9‐negative pancreatic cancer.

## 2 Patients and Methods

### 2.1 Patients

This retrospective study was conducted at the National Hospital Organization (NHO), Takasaki General Medical Center, and Saitama Medical University International Medical Center between May 2024 and July 2025. The study protocol was reviewed and approved by the institutional review board (approval no. TMG‐2025‐055). The requirement for written informed consent was waived due to the retrospective design. Informed consent was instead obtained using an opt‐out method via the institution's website and in‐hospital postings. The study was conducted in accordance with the ethical standards of the 1964 Declaration of Helsinki and its later amendments.

Patients who underwent abdominal computed tomography (CT) for the diagnosis of pancreatic disease and had their CEA, CA19‐9, and apoA2‐i indexes measured were included. The exclusion criteria were as follows: (1) patients with active malignancies other than pancreatic cancer; (2) patients with insufficient serum samples; and (3) patients with a large amount of missing clinical data. The collected data included age, sex, CEA, CA19‐9, apoA2‐i index, imaging findings (CT, MRI, and endoscopic ultrasound), pathological findings, stage of pancreatic cancer (UICC classification), tumor size, tumor location, presence of comorbidities, and the presence or absence of bile duct obstruction. Pancreatic lesions were clinically diagnosed using CT, MRI, and endoscopic ultrasound in accordance with the clinical practice guidelines of the Japan Pancreas Society [11]. For the diagnosis of pancreatic cancer, whenever the diagnosis was uncertain, a pathological diagnosis was made by endoscopic ultrasound‐guided fine‐needle biopsy (EUS‐FNB) or from surgical specimens. Pancreatic duct dilatation was defined as a maximum diameter of the main pancreatic duct of > 3 mm. Atrophic changes in the pancreatic parenchyma were categorized as none, mild to moderate, or severe based on enhanced CT scans [12]. The lesions were classified as pancreatic cancer, intraductal papillary mucinous neoplasm (IPMN), or other conditions such as chronic pancreatitis or autoimmune pancreatitis.

### 2.2 apoA2 Isoform Index and Definition

The concentrations of apoA2‐AT and apoA2‐TQ in serum samples were measured using an enzyme‐linked immunosorbent assay (TORAY APOA2‐iTQ; TORAY INDUSTRIES, INC., Tokyo, Japan). The apoA2‐i index was calculated as the geometric mean of the apoA2‐AT and apoA2‐TQ concentrations, as follows [9]:

$$\begin{array}{c} \text{apoA}2-i\;\text{index}=\sqrt{(\text{apoA}2-\mathrm{AT}\;\text{concentration}\times \text{apoA}2}\\ \;-\mathrm{TQ}\;\text{concentration}) \end{array}$$

If the apoA2‐AT concentration was < 3.25 μg/mL or the apoA2‐TQ concentration was < 5.75 μg/mL, the apoA2‐i index was defined as 0 μg/mL. The cutoff value for a positive apoA2‐i index was defined as < 59.5 μg/mL, consistent with the clinical reference value [10].

Serum concentrations of CEA and CA19‐9 were measured using chemiluminescent enzyme immunoassay kits (Elecsys CEA and Elecsys CA19‐9; Roche Diagnostics, Tokyo, Japan). The cutoff values were prespecified as 5.0 ng/mL for CEA and 37.0 U/mL for CA19‐9.

The sensitivity, specificity, positive predictive value (PPV), and negative predictive value (NPV) of CEA, CA19‐9, and the apoA2‐i index for the diagnosis of pancreatic cancer were calculated. Staging of pancreatic cancer was based on the TNM Classification of Malignant Tumors, 8th Edition [13]. Stages 0 and I were defined as early stage, and stages II–IV as advanced stage.

### 2.3 Statistical Analysis

Continuous variables were expressed as medians with interquartile ranges (IQRs). Categorical variables were analyzed using Fisher's exact test, and continuous variables were compared using the Mann–Whitney *U* test or the Kruskal–Wallis test for nonparametric unpaired data, and the Friedman test for nonparametric paired data. Statistical significance was defined as *p* < 0.05. Receiver operating characteristic (ROC) curves were constructed to evaluate diagnostic performance. All statistical analyses were performed using EZR version 1.68 (Saitama Medical Center, Jichi Medical University, Saitama, Japan) [14].

## 3 Results

### 3.1 Patient Characteristics

The characteristics of the patients are summarized in Table 1. A total of 115 patients were included in this study. The median age was 72.0 years (IQR, 62.0–77.0), and there were 55 males and 60 females. Diagnoses included 38 pancreatic cancers, 47 IPMNs, and 30 other conditions.

**TABLE 1:** Clinical characteristics of the patients.

Variables	All cases (*n* = 115)	Pancreatic cancer (*n* = 38)	IPMN (*n* = 47)	Others (*n* = 30)	*p*
Age (years), median (IQR)	72.0 (62.0–77.0)	74.0 (67.0–77.8)	71.0 (63.0–76.5)	69.5 (59.0–74.0)	0.2
Male, *n* (%)	55 (47.8)	22 (57.9)	22 (46.8)	11 (36.7)	0.2
CEA (ng/mL)	2.6 (1.6–3.8)	3.3 (2.1–5.2)	2.1 (1.6–3.2)	2.5 (1.3–3.1)	0.007
CEA (positive/negative)	17/98	10/28	4/43	3/27	0.11
CA19‐9 (U/mL)	12.9 (7.3–36.4)	58.8 (12.4–1367.2)	9.3 (5.3–14.3)	13.6 (7.2–23.3)	< 0.001
CA19‐9 (positive/negative)	29/86	21/17	2/47	6/24	< 0.001
apoA2 AT (μg/mL)	67.3 (28.6–99.5)	34.7 (4.0–72.2)	81.7 (44.9–102.5)	75.8 (56.1–112.3)	< 0.001
apoA2 TQ (μg/mL)	126.0 (82.6–166.0)	88.1 (39.4–150.5)	134.0 (109.5–171.0)	139.0 (117.0–169.8)	0.005
apoA2‐i index (μg/mL)	86.7 (47.8–106.0)	41.0 (2.2–72.2)	96.1 (75.9–118.5)	99.0 (75.7–129.0)	< 0.001
apoA2‐i index (positive/negative)	36/79	25/13	5/42	6/24	< 0.001
Pancreatic atrophy (non/mild/high/ND)	83/17/10/5	24/5/9/0	36/6/0/5	23/6/1/0	0.001
Dilation of pancreatic duct (Y/N)	42/73	22/16	12/35	8/22	0.004

Abbreviations: apoA2, apolipoprotein A2; apoA2‐i index, apolipoprotein A2 isoform index; CA19‐9, carbohydrate antigen 19‐9CEA; carcinoembryonic antigen; IPMN, intraductal papillary mucinous neoplasm; IQR, interquartile range.

Among patients with pancreatic cancer, serum levels of CEA (*p* = 0.007) and CA19‐9 (*p* < 0.001) were significantly higher, whereas the apoA2‐i index (*p* < 0.001) was significantly lower compared with those in the IPMN and other groups. The positivity rates of CA19‐9 and the apoA2‐i index were also significantly higher in patients with pancreatic cancer (*p* < 0.001), whereas the positivity rate of CEA did not differ significantly among the groups.

### 3.2 Diagnostic Performance of Tumor Markers

The diagnostic performance of CEA, CA19‐9, and the apoA2‐i index is shown in Table 2. Among the 115 patients, 17 (14.8%), 29 (25.2%), and 36 (31.3%) were positive for CEA, CA19‐9, and the apoA2‐i index, respectively. Forty‐six of the 115 patients (40.0%) were positive for either CA19‐9 or the apoA2‐i index.

**TABLE 2:** Diagnostic performance of three tumor markers for pancreatic cancer.

Variables		Sensitivity	Specificity	PPV	NPV
CEA	All	26.3% (10/38)	90.9% (70/77)	58.8% (10/17)	71.4% (70/98)
	Stage 0 or I	25.0% (2/8)	86.9% (93/107)	11.8% (2/17)	94.9% (93/98)
	Stage II–IV	26.7% (8/30)	88.2% (75/85)	47.1% (8/17)	76.5% (75/98)
CA19‐9	All	55.3% (21/38)	89.6% (69/77)	72.4% (21/29)	80.2% (69/86)
	Stage 0 or I	12.5% (1/8)	74.8% (80/107)	3.4% (1/29)	93.0% (80/86)
	Stage II–IV	66.7% (20/30)	88.3% (75/85)	69.0% (20/29)	87.2% (75/86)
apoA2‐i index	All	65.8% (25/38)	85.7% (66/77)	69.4% (25/36)	83.5% (66/79)
	Stage 0 or I	62.5% (5/8)	72.0% (77/107)	13.9% (5/36)	97.5% (77/79)
	Stage II–IV	66.7% (20/30)	81.2% (69/85)	55.6% (20/36)	87.3% (69/79)
Combination of CA19‐9 and apoA2‐i index	All	78.9% (30/38)	81.8% (63/77)	65.2% (30/46)	91.3% (63/69)
	Stage 0 or I	62.5% (5/8)	61.7% (66/107)	10.9% (5/46)	95.7% (66/69)
	Stage II–IV	83.3% (25/30)	75.3% (64/85)	54.3% (25/46)	92.8% (64/69)

Abbreviations: apoA2‐i index, apolipoprotein A2 isoform index; CA19‐9, carbohydrate antigen 19‐9; CEA, carcinoembryonic antigen; IPMN, intraductal papillary mucinous neoplasm; NPV, negative predictive value; PPV, positive predictive value.

**FIGURE 1:**
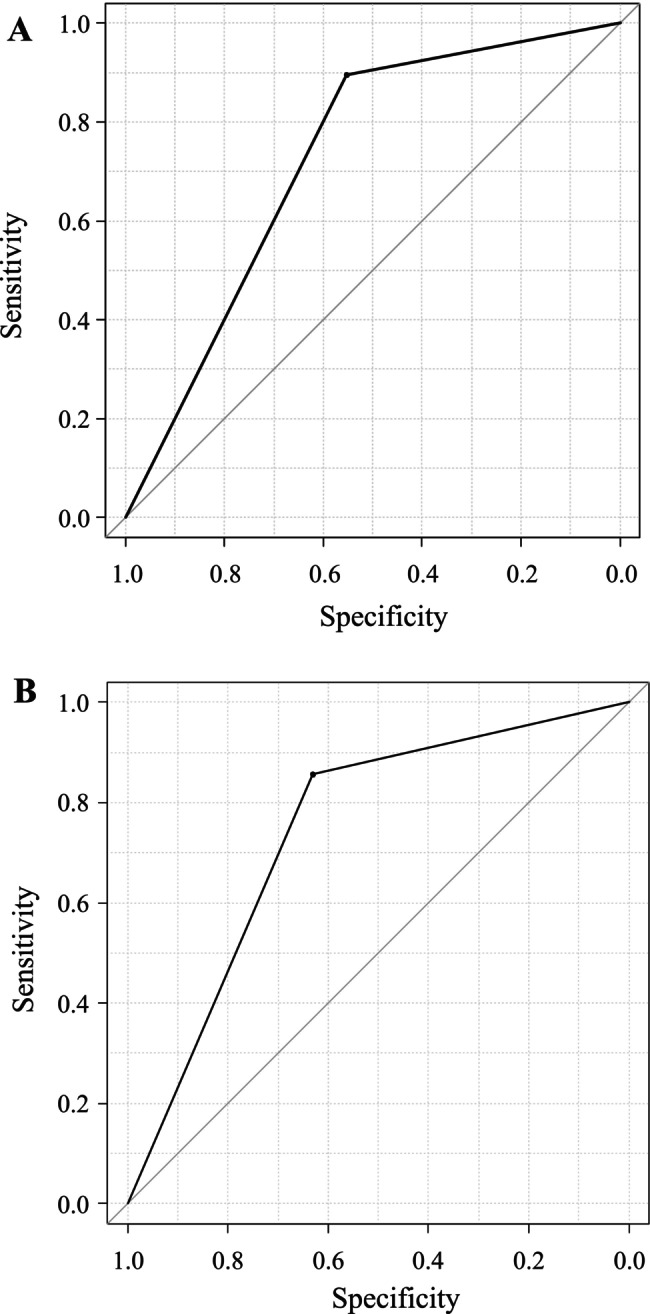
ROC curves for CA19‐9 and the apoA2‐i index. (A) The AUC of CA19‐9 was 0.724 (95% confidence interval: 0.637–0.812) when the cutoff value for CA19‐9 was set at 37.0 ng/mL. (B) The AUC of the apoA2‐i index was 0.744 (95% confidence interval: 0.657–0.831) when the cutoff value for the apoA2‐i index was set at 59.5 μg/mL. apoA2‐i index, apolipoprotein A2 isoform index; AUC, area under the curve; ROC, receiver operating characteristic.

The sensitivity, specificity, PPV, and NPV of CA19‐9 were as follows: All stages of pancreatic cancer, 55.3%, 89.6%, 72.4%, and 80.2%; stages 0/I, 12.5%, 74.8%, 3.4%, and 93.0%; and stages II–IV, 66.7%, 88.3%, 69.0%, and 87.2%. The ROC curve yielded an area under the curve (AUC) of 0.724 (95% confidence interval [CI], 0.637–0.812) (Figure 1A).

The sensitivity, specificity, PPV, and NPV of the apoA2‐i index were as follows: All stages of pancreatic cancer, 65.8%, 85.7%, 69.4%, and 83.5%; stages 0/I, 62.5%, 72.0%, 13.9%, and 97.5%; and stages II–IV, 66.7%, 81.2%, 55.6%, and 87.3%. The ROC curve yielded an AUC of 0.744 (95% CI, 0.657–0.831) (Figure 1B). There were no significant differences in sensitivity or specificity between the apoA2‐i index and CA19‐9.

The diagnostic performance for the combination of CA19‐9 and the apoA2‐i index is presented in Table 2. The sensitivity, specificity, PPV, and NPV were as follows: All stages of pancreatic cancer, 78.9%, 81.8%, 65.2%, and 91.3%; stages 0/I, 62.5%, 61.7%, 10.9%, and 95.7%; and stages II–IV, 83.3%, 75.3%, 54.3%, and 92.8%. The ROC curve yielded an AUC of 0.752 (95% CI, 0.669–0.835).

### 3.3 Comparison Between the Patients With CA19‐9‐Positive and ‐Negative Pancreatic Cancer

The clinical characteristics of patients with CA19‐9‐positive and ‐negative pancreatic cancers are shown in Table 3. There were 21 patients with CA19‐9–positive and 17 with CA19‐9‐negative pancreatic cancer. No significant differences were observed in clinical characteristics other than CA19‐9 titers and clinical stage. The proportion of early‐stage pancreatic cancers was significantly higher in the CA19‐9‐negative group (*p* = 0.03). Notably, 9 of 17 patients (52.9%) with CA19‐9‐negative pancreatic cancer were positive for the apoA2‐i index.

**TABLE 3:** Comparison of clinical characteristics between patients with CA19‐9‐positive and ‐negative pancreatic cancer.

Variables	Pancreatic cancer (*n* = 38)	CA19‐9‐positive (*n* = 21)	CA19‐9‐negative (*n* = 17)	*p*
Age (years), median (IQR)	74.0 (67.0–77.8)	75.0 (70.0–77.0)	72.0 (57.0–78.0)	0.2
Male, *n* (%)	22 (57.9)	12 (57.0)	10 (58.8)	1
CEA (ng/mL)	3.3 (2.1–5.2)	3.3 (2.7–6.3)	3.2 (2.0–4.5)	0.4
CA19‐9 (U/mL)	58.8 (12.4–1367.2)	1090.4 (167.0–3909.1)	10.7 (7.8–18.4)	< 0.001
apoA2 AT (μg/mL)	34.7 (4.0–72.2)	13.5 (3.8–45.7)	48.9 (4.7–90.4)	0.2
apoA2 TQ (μg/mL)	88.1 (39.4–150.5)	82.1 (42.4–165.0)	93.0 (38.8–134.0)	0.7
apoA2‐i index (μg/mL)	41.0 (2.2–72.2)	31.0 (8.9–55.6)	43.2 (0.0–95.0)	0.3
apoA2‐i index (positive/negative)	25/13	16/5	9/8	0.3
Pancreatic atrophy (non/mild/high)	24/5/9	14/2/5	10/3/4	0.9
Dilation of pancreatic duct (Y/N)	22/16	11/11	11/6	0.5
Clinical stages 0–I/II–IV	8/30	1/20	7/10	0.03

Abbreviations: apoA2, apolipoprotein A2; apoA2‐i index, apolipoprotein A2 isoform index; CA19‐9, carbohydrate antigen 19‐9; CEA, carcinoembryonic antigen; IQR, interquartile range.

### 3.4 Comparison Between the Patients With Stage 0/I and Stages II–IV Pancreatic Cancers

The clinical characteristics of patients with stage 0/I and stages II–IV pancreatic cancers are shown in Table 4. Eight patients had stage 0/I and 30 had stages II–IV pancreatic cancer.

**TABLE 4:** Clinical characteristics of patients with stage 0–I and stages II–IV pancreatic cancer.

Variables	Stages 0–I (*n* = 8)	Stages II–IV (*n* = 30)	*p*
Age (years), median (IQR)	72.0 (56.0–79.7)	74.5 (67.3–77.0)	0.6
Male, *n* (%)	4 (50.0)	18 (60.0)	0.4
CEA (ng/mL)	3.0 (1.3–4.7)	3.2 (2.4–6.3)	0.5
CA19‐9 (U/mL)	10.5 (8.3–23.5)	225.0 (22.4–2777.7)	0.02
CA19‐9 (positive/negative)	1/7	20/10	0.03
apoA2 AT (μg/mL)	35.0 (8.6–79.7)	45.7 (17.4–76.1)	0.9
apoA2 TQ (μg/mL)	110.0 (33.4–147.0)	93.0 (42.4–152.0)	0.4
apoA2‐i index (μg/mL)	32.6 (6.7–77.1)	45.5 (3.2–69.0)	0.6
apoA2‐i index (positive/negative)	5/3	20/10	1.0
Pancreatic atrophy (non/mild/high)	4/1/3	20/4/6	0.3
Dilation of pancreatic duct (Y/N)	5/2	17/14	0.2

Abbreviations: apoA2, apolipoprotein A2; apoA2‐i index, apolipoprotein A2 isoform index; CA19‐9, carbohydrate antigen 19‐9; CEA, carcinoembryonic antigen; IQR, interquartile range.

Among those with stage 0/I cancer, one of eight patients (12.5%) was positive for CA19‐9, whereas five of eight patients (62.5%) were positive for the apoA2‐i index. The CA19‐9 titer (*p* = 0.02) and positivity rate (*p* = 0.03) were significantly higher in advanced cancers than in early‐stage cancers. In contrast, neither the titer nor the positivity rate of the apoA2‐i index differed significantly between early and advanced stages. In other words, the positivity rate of the apoA2‐i index in early‐stage pancreatic cancer was comparable to that in advanced disease.

### 3.5 The Relation Between CT Findings and apoA2‐i Index

**FIGURE 2:**
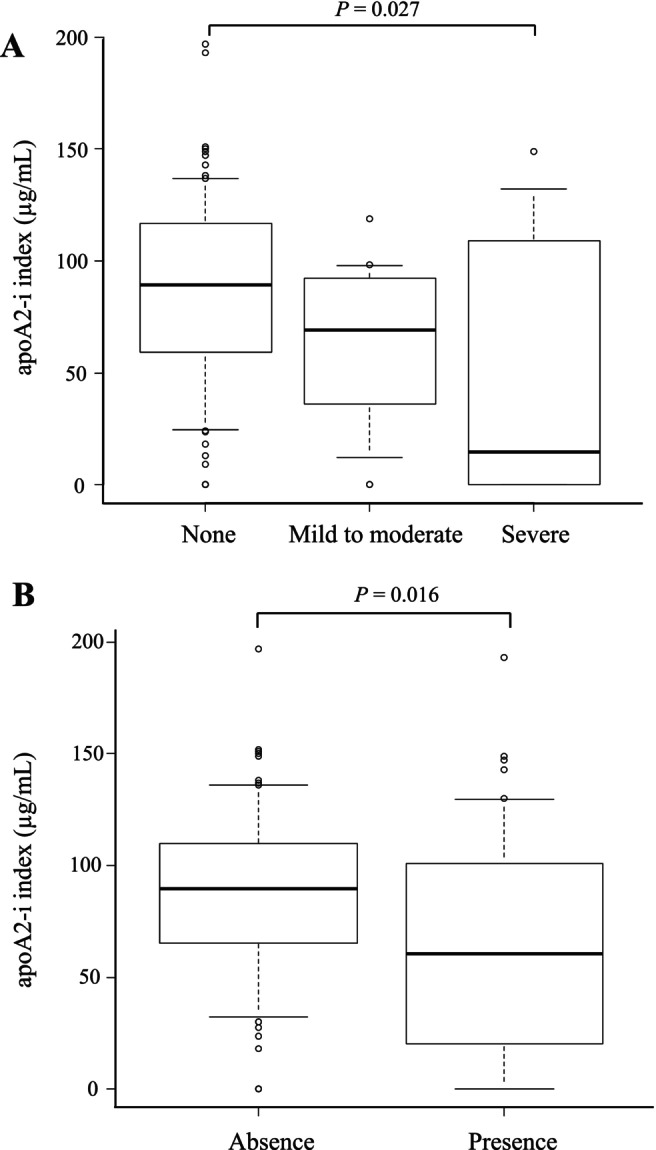
Relationship between computed tomography (CT) findings and the apoA2‐i index. (A) Relationship between pancreatic atrophy and the apoA2‐i index. The apoA2‐i index was significantly lower in patients with pancreatic atrophy (*p* = 0.027). (B) Relationship between pancreatic duct dilatation and the apoA2‐i index. The apoA2‐i index was significantly lower in patients with pancreatic duct dilatation (*p* = 0.016).

The relationship between pancreatic atrophy and the apoA2‐i index is shown in Figure 2A. The median apoA2‐i index in patients with no, mild to moderate, and severe pancreatic atrophy was 89.2 μg/mL (IQR, 59.3–116.5), 69.0 μg/mL (IQR, 36.0–92.4), and 14.6 μg/mL (IQR, 0.0–101.3), respectively. The apoA2‐i index significantly decreased with the progression of pancreatic atrophy (*p* = 0.027). The relationship between pancreatic duct dilatation and the apoA2‐i index is shown in Figure 2B.

The median apoA2‐i index in patients without pancreatic duct dilatation was 89.6 μg/mL (IQR, 65.1–110.0), whereas that in patients with duct dilatation was 60.6 μg/mL (IQR, 21.1–100.3). The apoA2‐i index was significantly lower in patients with pancreatic duct dilatation (*p* = 0.016).

## 4 Discussion

In this retrospective study, we demonstrated the clinical utility of the apoA2‐i index, a novel tumor marker for pancreatic cancer, particularly in cases that are negative for CA19‐9. The apoA2‐i index showed diagnostic performance comparable to that of CA19‐9, and the combination of both markers significantly improved the diagnostic sensitivity for pancreatic cancer. Importantly, more than half of the CA19‐9‐negative pancreatic cancer patients tested positive for the apoA2‐i index. Furthermore, there was no significant difference in the positive rate of the apoA2‐i index between early and advanced cancer, suggesting that it may be superior to CA19‐9 for detecting early‐stage disease.

Apolipoprotein A2 (apoA2) is one of the major components of high‐density lipoprotein (HDL) and is primarily synthesized in the liver and small intestine [15]. It circulates abundantly in plasma as a homodimer. The full‐length C‐terminal sequence is “A (alanine), T (threonine), Q (glutamine),” but several isoforms exist in which one or two amino acids are deleted from this region [16]. Among the five apoA2 homodimers with C‐terminal variants (TQ/TQ, AT/TQ, AT/AT, A/AT, and A/A), the three most prevalent in healthy individuals are TQ/TQ, AT/TQ, and AT/AT [16]. Honda et al. reported that the plasma concentration of AT/TQ dimers is reduced in patients with pancreatic cancer and developed a novel tumor marker, the apoA2‐i index, based on these findings [7, 8, 9, 10]. The apoA2‐i index primarily reflects pancreatic exocrine dysfunction caused by pancreatic cancer and thus serves as a biomarker for early detection [7, 8, 9, 10]. The differences in amino acids at the C‐terminus of each isoform are believed to result from cleavage by pancreatic exopeptidases such as carboxypeptidase A [7, 8, 9, 10]. In patients with pancreatic cancer, pancreatic exocrine dysfunction leads to aberrant processing of apoA2 dimers, resulting in a significant reduction of the apoA2‐AT/TQ isoform [7, 8, 9, 10]. In a previous study, the AUC of the apoA2‐i index (0.879) was higher than that of CA19‐9 (0.849) [10]. Similarly, in our study, the AUC of the apoA2‐i index was slightly higher than that of CA19‐9.

The apoA2‐i index has been clinically available in Japan since March 2024. Shinomiya et al. [17] recently reported its usefulness for the diagnosis of pancreatic cancer in real‐world clinical practice. They found that while the apoA2‐i index had lower diagnostic accuracy for advanced (Stages II–IV) pancreatic cancer compared with CA19‐9, it provided superior accuracy for early‐stage (stage 0/I) detection. Consistently, our study showed that the detection rate of the apoA2‐i index did not significantly differ between early and advanced cancers and was superior to CA19‐9 for early detection.

CA19‐9 is detected using a monoclonal antibody that recognizes a carbohydrate structure known as the sialylated Lewis *a* antigen [18]. This carbohydrate epitope is synthesized by enzymes involved in the formation of Lewis blood group antigens (Lewis *a/b*) on red blood cells. Therefore, individuals who are genetically Lewis antigen‐negative (Le[a−b−]) do not produce CA19‐9, and its serum levels remain undetectable even in the presence of pancreatic cancer [19, 20]. Such CA19‐9‐negative pancreatic cancers occur in approximately 5%–10% of the population [19, 20]. In contrast, the apoA2‐i index is not affected by Lewis antigen status. In the present study, 52.9% of patients with CA19‐9‐negative pancreatic cancer were positive for the apoA2‐i index. These cases often included patients with early‐stage or well‐differentiated pancreatic adenocarcinoma. Indeed, in our cohort, CA19‐9‐negative pancreatic cancer was more frequently observed in early‐stage (stage 0/I) disease. The ability of the apoA2‐i index to identify more than half of these patients suggests its potential value in screening and diagnostic algorithms.

The apoA2‐i index was also associated with progression of chronic pancreatitis and with morphological changes observed on CT, including pancreatic atrophy and main pancreatic duct dilatation [21]. These findings are consistent with the proposed biological mechanism of this biomarker. The apoA2‐i index reflects decreased secretion of normal apoA2 dimers due to pancreatic exocrine dysfunction or destruction of acinar cells by the tumor [7, 8, 9, 10]. Consequently, a lower apoA2‐i index can serve as a surrogate marker for pancreatic exocrine insufficiency. Pancreatic atrophy and ductal dilatation are well‐recognized imaging features of both early and advanced pancreatic cancer and are often used as indirect diagnostic clues [22]. The cross‐reactivities of apoA2‐i for gastric, colorectal, and liver cancers were lower than those of CA19‐9 [10]. However, because these cross‐reactivity results were obtained from a small sample size, validation studies using large real‐world datasets from clinical settings will be needed [10].

The combination of CA19‐9 and the apoA2‐i index achieved the highest diagnostic performance, increasing sensitivity to 78.9% while maintaining acceptable specificity (81.8%). This complementary relationship is clinically meaningful. A diagnostic algorithm integrating both CA19‐9 and the apoA2‐i index may improve early detection and facilitate the selection of patients for further imaging or tissue biopsy.

This study has several limitations. First, the sample size was relatively small, particularly for stage 0/I pancreatic cancer, which limited the statistical power of subgroup analyses. Second, the retrospective design may have introduced selection bias, as only patients who underwent simultaneous measurement of CEA, CA19‐9, and the apoA2‐i index were included. Further large‐scale, prospective studies focusing on stage 0/I and CA19‐9‐negative pancreatic cancers are warranted to validate our findings.

In conclusion, the apoA2‐i index is a useful tumor marker for pancreatic cancer, independent of Lewis antigen status. It demonstrated diagnostic accuracy comparable to that of CA19‐9 and may be superior for the early detection of pancreatic cancer.

## Funding

The authors have nothing to report.

## Conflicts of Interest

The authors declare no conflicts of interest.

## Data Availability

The data that support the findings of this study are available from the corresponding author upon reasonable request.
