# Correction to “Clinical Utility of the Apolipoprotein A2 Isoform Index as a Tumor Marker for Pancreatic Cancer”

**DOI:** 10.1002/jgh3.70431

**Published:** 2026-06-08

T. Hoshino , M. Mizuide , Y. Suzuki , H. Yasuoka , A. Naganuma , T. Hatanaka , S. Kakizaki , S. Ryozawa , “Clinical Utility of the Apolipoprotein A2 Isoform Index as a Tumor Marker for Pancreatic Cancer,” JGH Open 10, no. 3 (2026): e70375, 10.1002/jgh3.70375.41767619 PMC12946653

In the published version, the second affiliation for the first author, Takashi Hoshino, was inadvertently omitted.

The correct affiliations for the first author should be as follows:

Takashi Hoshino^1,2^

^1^Department of Gastroenterology, NHO Takasaki General Medical Center, Takasaki, Japan

^2^Department of Gastroenterology, Saitama Medical University International Medical Center, Hidaka, Japan

We apologize for this error.

